# Requirement of *Toxoplasma gondii* metacaspases for IMC1 maturation, endodyogeny and virulence in mice

**DOI:** 10.1186/s13071-021-04878-0

**Published:** 2021-08-12

**Authors:** Muzi Li, Jing Liu, Yayun Wu, Yihan Wu, Xiaodong Sun, Yong Fu, Xiao Zhang, Qun Liu

**Affiliations:** 1 National Animal Protozoa Laboratory, College of Veterinary Medicine, China Agricultural University, Beijing, China; 2 Key Laboratory of Animal Epidemiology of the Ministry of Agriculture, College of Veterinary Medicine, China Agricultural University, Beijing, China; 3 China Animal Health and Epidemiology Center, Qingdao, Shandong China

**Keywords:** Metacaspases, Inner membrane complex 1, Maturation, Endodyogeny, *Toxoplasma gondii*

## Abstract

**Background:**

Metacaspases are multifunctional proteins found in plants, fungi and protozoa, and are involved in processes such as insoluble protein aggregate clearance and cell proliferation. Our previous study demonstrated that metacaspase-1 (MCA1) contributes to parasite apoptosis in *Toxoplasma gondii*. Deletion of MCA1 from *T. gondii* has no effect on the growth and virulence of the parasites. Three metacaspases were identified in the ToxoDB *Toxoplasma* Informatics Resource, and the function of metacaspase-2 (MCA2) and metacaspase-3 (MCA3) has not been demonstrated.

**Methods:**

In this study, we constructed MCA1, MCA2 and MCA1/MCA2 transgenic strains from RHΔku80 (Δku80), including overexpressing strains and knockout strains, to clarify the function of MCA1 and MCA2 of *T. gondii*.

**Results:**

MCA1 and MCA2 were distributed in the cytoplasm with punctuated aggregation, and part of the punctuated aggregation of MCA1 and MCA2 was localized on the inner membrane complex of *T. gondii*. The proliferation of the MCA1/MCA2 double-knockout strain was significantly reduced; however, the two single knockout strains (MCA1 knockout strain and MCA2 knockout strain) exhibited normal growth rates as compared to the parental strain, Δku80. In addition, endodyogeny was impaired in the tachyzoites whose MCA1 and MCA2 were both deleted due to multiple nuclei and abnormal expression of IMC1. We further found that IMC1 of the double-knockout strain was detergent-soluble, indicating that MCA1 and MCA2 are associated with IMC1 maturation. Compared to the parental Δku80 strain, the double-knockout strain was more readily induced from tachyzoites to bradyzoites in vitro. Furthermore, the double-knockout strain was less pathogenic in mice and was able to develop bradyzoites in the brain, which formed cysts and established chronic infection.

**Conclusion:**

MCA1 and MCA2 are important factors which participate in IMC1 maturation and endodyogeny of *T. gondii*. The double-knockout strain has slower proliferation and was able to develop bradyzoites both in vitro and in vivo.

**Graphic abstract:**

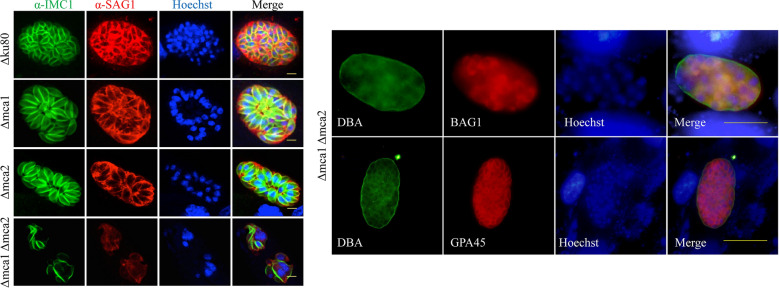

**Supplementary Information:**

The online version contains supplementary material available at 10.1186/s13071-021-04878-0.

## Background

*Toxoplasma gondii* tachyzoites, as single-celled eukaryotes, undergo binary divisions, including karyokinesis and cytokinesis, in a process termed endodyogeny [1–3], where two daughter cells are assembled simultaneously within one mother cell using a scaffold known as the inner membrane complex (IMC) [4]. The IMC is a highly specialized endomembrane system that lies directly beneath the plasma membrane and plays several important roles in the complex life cycle of the parasite, including providing structural stability, serving as an important scaffold in daughter cell development and anchoring of the actin-myosin motor complex, which is a key component of parasite motility and host cell invasion [5]. At the onset of daughter cell formation, new IMCs assemble within the cytoplasm and elongate rapidly, coordinating the segregation of subcellular organelles according to a strict schedule [4].

IMC formation undergoes a process that includes initiation, elongation, emergence and maturation, while some are synthesized de novo, using some components recycled from the maternal cells [6, 7]. The IMC is composed of alveoli, which are a kind of flattened membrane sac, and the function of the alveoli has not been clearly defined except as an anchor for IMC-resident proteins. The subpellicular network (SPN) beneath the alveoli consists of interwoven 8–10 nm filaments, which confer strength and stability to the parasite. These filaments are also known as alveolins, and the first alveolin identified by Apicomplexa researchers was IMC1 (inner membrane complex 1) localized to the SPN [8]. Some researchers found that the SPN of immature parasites were completely soluble in detergents such as deoxycholate and Triton X-100, but the network of mature parasites was entirely detergent-resistant [9]. Moreover, *Tg*IMC1 of immature parasites was reported to be highly soluble in detergents, whereas *Tg*IMC1 of mature parasites was not. The conversion of IMC1 from a detergent-soluble network to a detergent-resistant network coincides with the proteolytic removal of the carboxyl terminus of *Tg*IMC1. At later stages of daughter cell maturation, a 5 kDa peptide is removed from the C-terminus of IMC1 by a proteolytic process, allowing for the maturation of IMC1 with greater stability and mechanical strength to maintain cell shape and integrity [9].

*Toxoplasma gondii* expresses many cysteine proteases, such as cathepsin proteases, which are critical for the growth and survival of the parasite [10, 11]. We previously demonstrated that a cysteine protease, metacaspase-1, participated in the apoptosis of *T. gondii* [12]. Metacaspases are cysteine proteases belonging to the clan CD, family C14, and are orthologs of both caspases and paracaspases [13, 14]. Metacaspases are apoptosis-like executioner proteins in plants, fungi and protozoan parasites [15, 16]. In addition to caspase-like activity, metacaspases are one factor in the quality control of budding yeast proteins [17, 18]. Metacaspases also have a role in cell cycle progression, as manipulation of the transcription level of metacaspases leads to arrest or delay of cell karyokinesis and cytokinesis in *Leishmania* [14, 19, 20]. In a previous study, we found that overexpression of metacaspase-1 impaired the growth of tachyzoites [12], implying that metacaspases are important in *T. gondii* proliferation. To verify the hypothesis that metacaspases (MCAs) regulate the cell cycle of tachyzoites, we constructed an MCA2 knockout strain (Δmca2) and an MCA1/MCA2 double-knockout strain (Δmca1Δmca2) from Δku80 and demonstrated the requirement of MCA1 and MCA2 for IMC1 maturation, proliferation and virulence of *T. gondii*.

## Methods

### Parasite maintenance and cell culture

Human foreskin fibroblast (HFF) and Vero cells were cultured in Dulbecco’s modified Eagle’s medium (DMEM) containing 25 mM glucose and 4 mM glutamine supplemented with 10% fetal bovine serum (FBS, Gibco, USA) as described previously [12]. *Toxoplasma gondii* RHΔku80 tachyzoites were maintained in vitro by serial passages on confluent Vero cells at 37 °C with 5% CO_2_ in a humidified incubator. The *Tg*MCA1 knockout strain (Δmca1) [12] was incubated under the same conditions as *T*. *gondii* RHΔku80.

### Western blot and immunofluorescence assays

Freshly isolated parasites were harvested and purified by filtration through a 5 µm filter, and were collected by centrifugation at 1400×*g* for 10 min and washed in phosphate-buffered saline (PBS). The purified parasites were lysed in RIPA buffer (Beyotime, Shanghai) with the protease inhibitor PMSF (Beyotime, Shanghai). The lysate (7–10 µg) was subjected to SDS-PAGE (8% w/v for MCA1 and 6% w/v for MCA2) electrophoresis and transferred to polyvinylidene fluoride (PVDF) membranes (Millipore, USA). The membranes were sealed with 5% (w/v) skim milk in PBS and then incubated for 1 h at room temperature with mouse anti-r*Tg*MCA1/2 antibody at 1:500 dilution in 5% skim milk in PBS, followed by goat anti-mouse IgG (H+L) horseradish peroxidase (Sigma-Aldrich, USA) as secondary antibody at 1:5000 dilution in 5% skim milk in PBS. Finally, appropriate chemiluminescent reagents (CoWin Biotech Co., Ltd., Beijing) were used for reactive band visualization.

Immunofluorescence assays (IFA) for TgMCA1 and TgMCA2 subcellular localization were performed as described previously [12]. Prior to the IFA test, transgenic tachyzoites overexpressing MCA1 or MCA2 were prepared. The parasites were seeded onto HFFs on glass coverslips in 12-well plates. Infected cells were incubated at 37 °C with 5% CO_2_ for no more than 20 h and fixed for 15 min in 4% formaldehyde, after which they were permeabilized with 0.25% Triton X-100 for 15 min and blocked with 3% bovine serum albumin (BSA) for 30 min. Subsequently, the cells were incubated with primary antibody (mouse anti-HA monoclonal antibody, Sigma-Aldrich, USA), mouse anti-*Tg*IMC1 polyclonal antibody at 1:500 dilution, mouse anti-*Tg*GAP45 polyclonal antibody at 1:500 dilution, mouse anti-*Tg*GRA1 polyclonal antibody at 1:100 dilution) at 37 °C for 1 h, followed by FITC-conjugated goat-anti mouse IgG (H+L) (Sigma-Aldrich, USA) at 1:100 dilution with 3% BSA at 37 °C for 1 h. The nucleus was stained with Hoechst 33258 (Sigma-Aldrich, USA) for 5 min. Rabbit anti-*Tg*SAG1 antibody serum was used as a control. The parasites were observed and images were obtained using a Leica confocal microscope system (Leica TCS SP52, Germany). Images were processed using LAS AF Lite 2.2.0 software.

### Generation of MCA mutant strains

The parental strain used to generate the knockout mutants was RHΔku80 (Δku80). Briefly, approximately 2000 bp of the 5′ flanking and 3′ flanking sequences of *Tg*MCA2 were amplified from the Δku80 genome. PDHFR *Tg*MCA2 KO was used to disrupt the native loci of the Δku80 tachyzoites by double homologous recombination and replacement of the entire coding region of *Tg*MCA2, and stable clones were derived by pyrimethamine selection. The primers were designed to identify the correct clones, which were also confirmed by western blot. Meanwhile, the *Tg*MCA2 complete coding sequence under the *Tg*GRA1 promoter was transfected into Δku80 and the parasites were selected by pyrimethamine. To further characterize the role of *Tg*MCAs, we generated a strain lacking both *Tg*MCA1 and *Tg*MCA2 (Δmca1Δmca2) based on Δmca1. The double-knockout parasites were screened under the pressure of pyrimethamine and chloramphenicol. Clones were identified by PCR and western blot as described above.

### Plaque assay

The plaque assay was performed on HFF cells cultured in six-well plates (Corning Costar, USA) as previously described [12]. A total of 500 freshly isolated parasites were seeded into HFF monolayers and incubated at 37 °C with 5% CO_2_ for 7 days without disturbance. After 7 days, the medium was removed from the HFFs and the cells were washed five times with PBS. Cell monolayers were fixed with 4% formaldehyde for 10 min, stained with 0.2% crystal violet solution for 30 min, washed with deionized water, and then visualized by microscopy (Olympus Co., Japan). The six-well plates were scanned using a Canon digital scanner (F917500, Japan).

### Intracellular parasite replication assay

Freshly isolated parasites (1 × 10^6^) were inoculated on HFFs in 12-well plates. After 30 min, the extracellular parasites were removed by washing with PBS. After 24 h of incubation, infected cells were fixed with 4% formaldehyde, and parasites were stained with rabbit anti-*T. gondii* positive serum following the IFA protocol. The number of parasites per vacuole was counted using a fluorescence microscope (Olympus Co., Japan) at ×400 magnification, and the number of tachyzoites per vacuole was scored with a total of 100 parasitophorous vacuoles (PV) for each strain. Data were analyzed by three independent experiments.

### Virulence assay in mice

The virulence assay was performed as described previously [12]. Eight-week-old female BALB/c mice were purchased from the Laboratory Animal Center of the Military Academy and acclimatized for 7 days prior to the experiment. Animals were maintained under specific pathogen-free conditions and provided with rodent chow and water. The parasites were injected intraperitoneally into mice at a dose of 100 tachyzoites (5 mice per parasite strain) and monitored for clinical signs and mortality every 8 h. Mice were humanely euthanized by cervical dislocation when they had no access to food or water for more than 24 h or lost 20% of their body weight. Survival data were compiled from three independent experiments.

### Detergent extraction of TgIMC1

HFF cells were prepared in 25 cm culture flasks, and were infected with 1 × 10^6^ Δmca1, Δmca2 and Δku80, and 1 × 10^7^ Δmca1Δmca2. After 16–20 h, HFF cells were washed twice with cold TBS and scraped into 2 ml of TBS. The parasites were collected by centrifugation at 600×*g* for 5 min and purified by filtration through a 5 µm filter. The collected parasites were then lysed on ice in 1% DOC in TBS for 5 min in the presence of protease inhibitors. DOC-soluble and insoluble materials were prepared by centrifugation at 27,400×*g* for 10 min. The DOC-insoluble pellet was re-extracted with DOC as above, and the DOC-soluble fractions were pooled. The pellet and soluble fractions were analyzed by SDS-PAGE on a 6% gel and immunoblotted with anti-*Tg*IMC1 antibodies at a 1:1000 dilution.

### Cyst induction by alkalized culture medium

The cyst induction medium was prepared with 1.35% DMEM without sodium bicarbonate, 1 M HEPES, 5% serum and 1% antibiotics, and the pH was adjusted to 8.2 with KOH. The medium was stored at 4 °C before use. Freshly isolated parasites (1 × 10^5^) were inoculated onto HFFs in 12-well plates in cell culture medium at 37 °C with 5% CO_2_ for 2 h. The medium was then replaced with cyst induction medium and incubated at 37 °C without CO_2_ for 4 days. After fixation in 4% formaldehyde for 15 min, the cells were permeabilized with 0.25% Triton X-100 for 15 min and then blocked with 3% bovine serum albumin (BSA) for 30 min. Cells were then incubated with dolichos biflorus agglutinin (DBA), mouse anti-*Tg*BAG1, or anti-*Tg*GAP45 antibody diluted 1:50 at 37 °C for 1 h, as well as secondary antibody and Hoechst 33258. Coverslips were observed and images were obtained using a Leica confocal microscope system (Leica TCS SP52, Germany). Images were processed using LAS AF Lite 2.2.0 software, and the rate of bradyzoite formation was calculated by dividing the number of PVs of DBA-positive parasites by the total number of PVs of anti-*Tg*GAP45 serum-labeled parasites. Data were obtained from three independent experiments, each in triplicate, and a total of 100 PVs were counted in each well.

### Transmission electron microscopy

Electron microscopy samples were performed by the Institute of Atomic Energy Utilization, Chinese Academy of Agricultural Sciences, and processed using routine techniques, summarized as follows: the parasites were incubated in Vero at 37 °C for 30 h and collected by centrifugation. Pellets were fixed in 2.5% glutaraldehyde in 0.1 M phosphate buffer, post-fixed in osmium tetroxide, dehydrated in ethanol and treated with propylene oxide, before being embedded in Spurr’s epoxy resin. Thin sections were stained with uranyl acetate and citrate. Samples were recorded with a Hitachi S-3400N scanning electron microscope (Shimadzu, Japan).

### Statistical analysis

Statistical tests for virulence, intracellular parasite replication and the rate of cyst induction were performed using SAS (SAS Institute Inc., USA). Two-way ANOVA was used to analyze the parasite replication data, and the chi-square was used to analyze each two parasite lines. The virulence of the strains to mice were determined used the life span test. The rate of bradyzoite induction in vitro was analyzed via the *t*-test. In all cases, the two-tailed *P*-value was calculated and differences were considered significant if the *P*-value was ≤ 0.05.

## Results

### Analysis of MCAs and their localization

Three ICE family protease (caspase) P20 domain-containing proteins were identified in the ToxoDB *Toxoplasma* Informatics Resource by BLAST using the amino acid sequences of metacaspases from *Saccharomyces cerevisiae*. They were named MCA1 (TGGT1_206490), MCA2 (TGGT1_278975) and MCA3 (TGGT1_243298) in our study. The transcriptional levels of MCA1, MCA2 and MCA3 are extremely low (see ToxoDB website), and the FPKM values in tachyzoites are 1/100th of the surface antigen protein 1 (SAG1) values and 1/1000th of the dense granule protein 1 (GRA1) values. However, the CRISPR Screen Value was −2.74 for MCA1, −3.93 for MCA2, and −2.97 for MCA3 (these values are shown on the ToxoDB website), indicating that all three genes are functionally important to parasites.

**Fig. 1:**
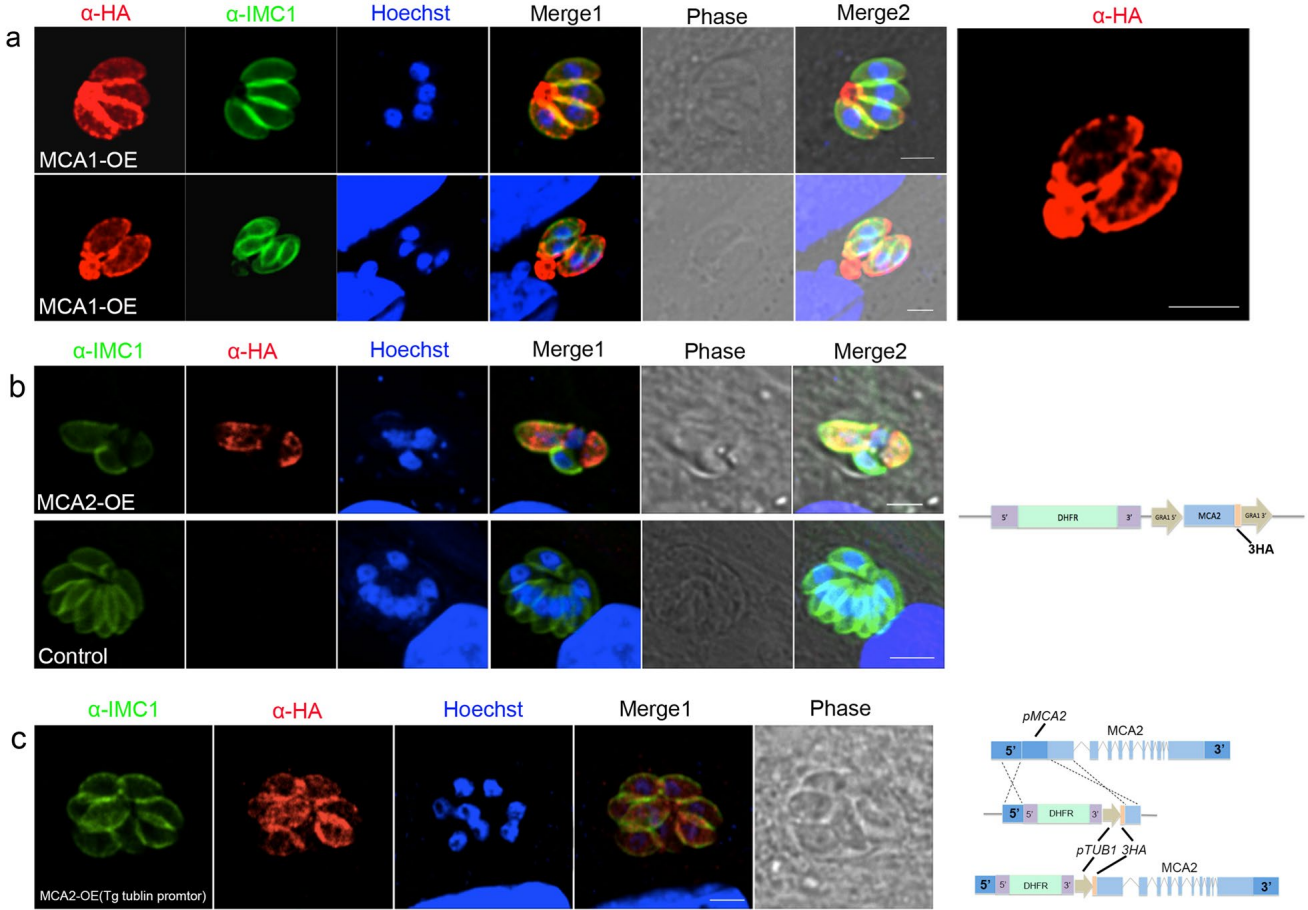
Localization of MCA1 and MCA2. **a** Localization of MCA1 in a *Toxoplasma gondii* strain with transient MCA1 overexpression (MCA1-OE). The top and bottom panels showed MCA1 tagged with HA was distributed in the cytoplasm of *T. gondii*, and was localized in the membrane of tachyzoites as punctate aggregates using anti-HA antibody. MCA1 localized at the IMC as the co-localization of MCA1 with IMC1 were found. The scale bar is 5 μm. **b** Tachyzoites with MCA2 overexpression using GRA1 promoter was confirmed to fail to multiply (shown as the top panel, MCA2-OE), and the growth of parasites which were not overexpressing MCA2 was normal (shown as the bottom panel, control). *Tg*MCA2 coding sequence tagged with HA at the C-terminus driven by the GRA1 promoter was randomly inserted to the genome of the parasites (as shown in the schematic of the experimental design). The scale bar is 5 μm. **c** Localization of MCA2 in a *T. gondii* strain with transient MCA2 overexpression under *Tg*Tubulin promoter (MCA2-OE under *Tg*Tubulin, as shown in the schematic of the experimental design). MCA2 with HA tag controlled by *Tg*Tubulin promoter was distributed in cytoplasm and partially co-localized with IMC1. The scale bar is 5 μm

In this study, we generated parasites that transiently express MCA1-HA to further confirm the localization of MCA1. Consistent with our previous findings using *Tg*MCA1 antiserum, we observed that MCA1 was distributed in the cytoplasm as discrete spots using an anti-HA monoclonal antibody (Fig. 1a). Surprisingly, MCA1 was localized not only in the cytoplasm but also on the IMC, with some punctate aggregation of *Tg*MCA1 co-localized with *Tg*IMC1 (Fig. 1a). These findings suggest that MCA1 may be associated with the function of the IMC.

We have also attempted to generate a mutant overexpressing MCA2-HA driven by the GRA1 promoter to obtain localization of MCA2. However, the mutant failed to proliferate (Fig. 1b), making us believe that overexpression of *Tg*MCA2 is lethal to the parasite. We then used the comparatively moderate *Tg*Tubulin1 promoter to target the native promoter of *Tg*MCA2 in Δku80. IFA showed that MCA2 was highly expressed and distributed in the cytoplasm and was also partially co-localized with IMC1 (Fig. 1c). Notably, the *Tg*MCA2 overexpression mutant under the tubulin promoter may be lost when cultured in vitro, despite the fact that we could observe the localization of *Tg*MCA2 using first-generation parasites after transfection. We believe that the failure of the construction of *Tg*MCA2 overexpression strains is associated with excess MCA2, which leads to parasite defects or death.

### Parasites lacking both MCA1 and ΜCA2 remain viable in vitro, but proliferation appears to be significantly impaired

We previously generated a complete *Tg*MCA1 knockout mutant (Δmca1) of the Δku80 strain and demonstrated that MCA1 is involved in the apoptotic pathway, but does not affect the growth and virulence of the parasites. To further evaluate the function of *Tg*MCAs, we generated a MCA1/MCA2 double-knockout mutant. MCA2 in the Δmca1 mutant was replaced by DHFR (dihydrofolate reductase) and was screened by pyrimethamine. Replacement of the endogenous MCA2 locus was confirmed by genomic PCR using the indicated oligonucleotides (Additional file 1: Figure S1a). Western blot was carried out to determine *Tg*MCA2 expression in the knockout strains (Additional file 1: Figure S1b) and to confirm that the MCA2 gene was completely deleted.

**Fig. 2:**
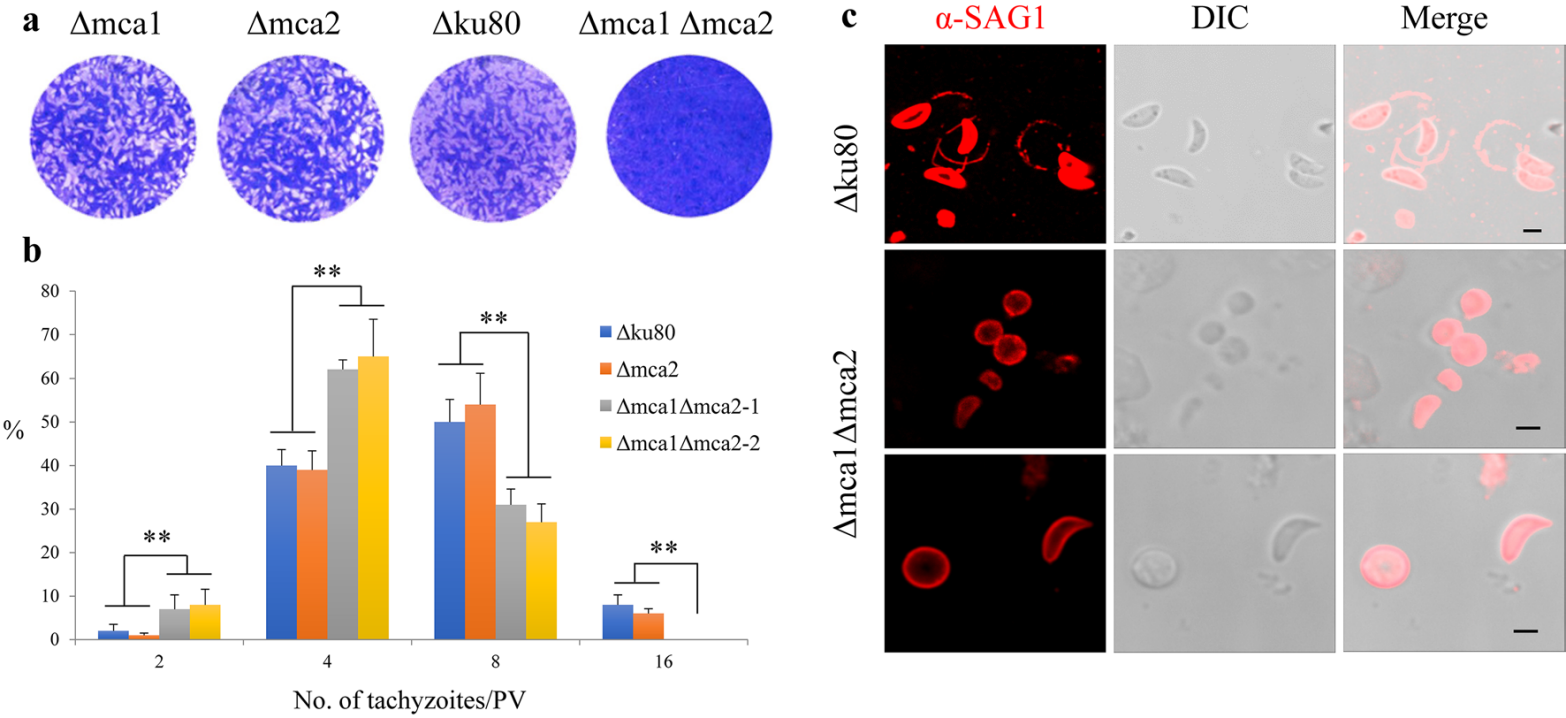
Knockout of MCA1 and MCA2 affected the growth of the tachyzoites. **a** Plaque assay of Δmca1, Δmca2, Δmca1Δmca2, and Δku80 tachyzoites. Each well of HFF was infected with 500 parasites and plaques were stained 7 days later. The plaque of Δmca1Δmca2 was not obvious compared to the other strains, which explained the defective proliferation of double-knockout strain. **b** Intracellular replication assay of Δmca1, Δmca2, Δmca1Δmca2, and Δku80 strains. A total of 100 PVs of each strain were counted, and data compiled from three independent assays were analyzed. *X*-axis is the number of tachyzoites in each PV. *Y*-axis is the percentage of PVs with different number of tachyzoites. *P < 0.5 was significant by the chi-square test. **c** Motility assay of Δmca1Δmca2 and Δku80. The motility was observed using IFA as the surface protein SAG1 was left behind with the motion of the parasites. The motility of Δku80 (top panel) was normal for most of the parasites had gliding and cycling motion. However, the double-knockout strain Δmca1Δmca2 (middle and bottom panel) showed abnormal morphology, with almost round shape of the extracellular parasites, and the abnormal extracellular parasites showed defective motility without any gliding and cycling motion. The scale bar from top to bottom panel: 2.5 μm, 2 μm and 3 μm

The plaque assay showed a severe proliferation defect in Δmca1Δmca2 compared to the two single knockout strains and Δku80 (Fig. 2a), as no obvious plaques were formed after 7 days in HFF. The intracellular replication of Δmca1Δmca2 was impaired as shown by calculating the number of parasites per vacuole after 24 h of incubation compared to the number of tachyzoites in each PV of Δmca2 and Δku80 (Fig. 2b). Furthermore, we observed an interesting phenomenon that most extracellular Δmca1Δmca2 tachyzoites were abnormally distorted and almost circular, and we found that distorted and circular parasites were defective in parasite motility, with less obvious gliding and circling paths (Fig. 2c).

### Association of MCA1 and MCA2 with tachyzoite maturation

**Fig. 3:**
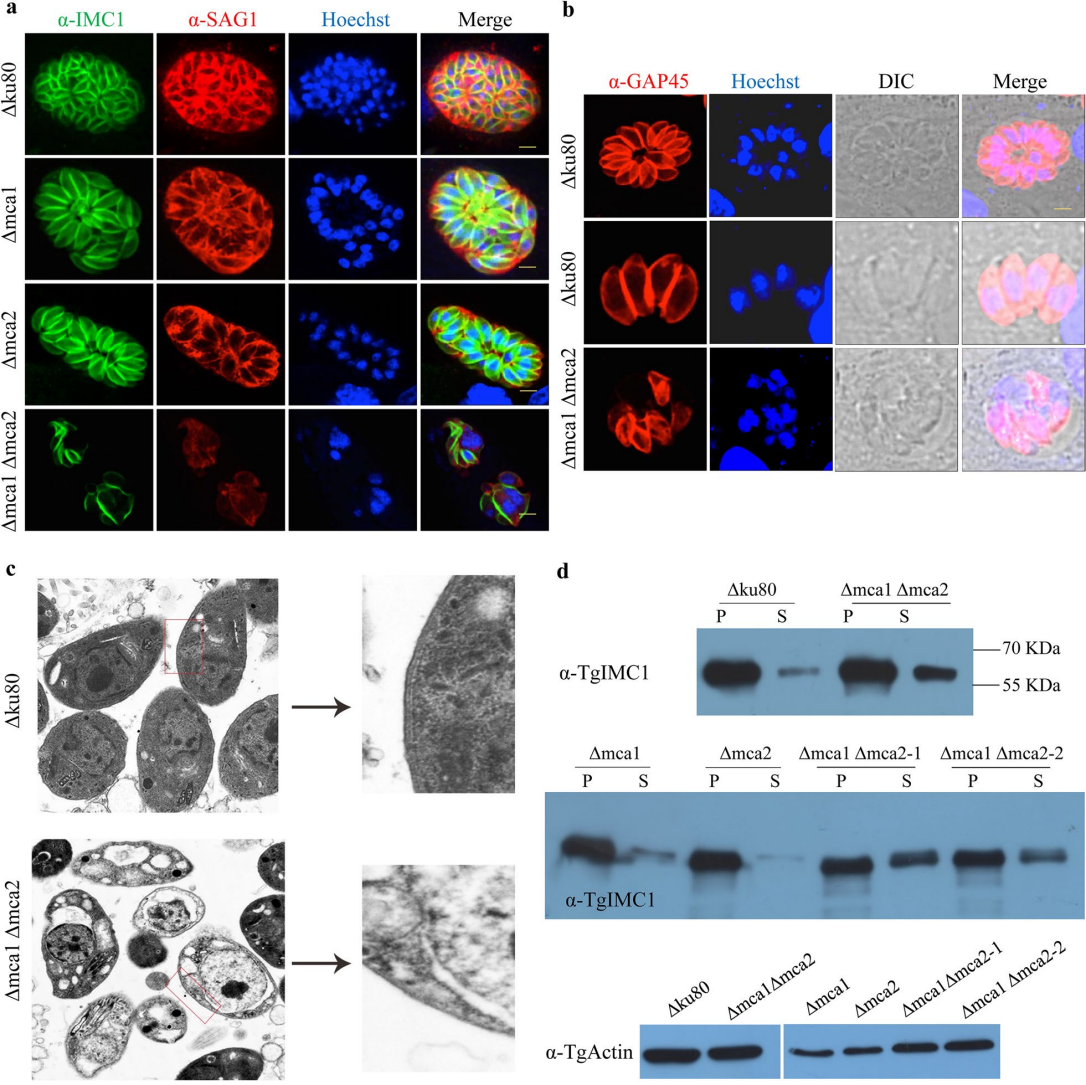
Tachyzoites lacking both MCA1 and MCA2 had defective maturation of IMC1. **a** Localization of IMC1 by IFA using anti-*Tg*IMC1 polyclonal antibody and anti-*Tg*SAG1 polyclonal antibody. Δmca1Δmca2 showed abnormal endodyogeny compared to Δku80, Δmca1 and Δmca2 cultured in HFF for 30 h. Scale bar from top to bottom panel: 10 μm, 5 μm, 5 μm and 5 μm. **b** Expression and localization of GAP45 were also abnormal as the same as that of IMC1 in Δmca1Δmca2. Scale bar from top to bottom panel: 2.5 μm, 5 μm, 5 μm. **c** Observation of *Toxoplasma gondii* by transmission electron microscopy. Δku80 was appeared to have normal nucleus and cytoplasm, while Δmca1Δmca2 showed many vacuoles in the cytoplasm. The right panel shows the outer membrane of Δku80 and Δmca1Δmca2. Δku80 showed clearly three layers of the outer membrane, while there are no distinguishable layers in Δmca1Δmca2. **d** Detergent resistance of IMC1 in Δmca1Δmca2 by western blot using DOC extraction. Western blot showed that IMC1 was both in the sediment and supernatant after total parasite extraction by DOC. IMC1 of Δmca1Δmca2 was more detergent-soluble than Δmca1, Δmca2 and Δku80, indicating that MCA1 and MCA2 are involved in IMC1 maturation. *Tg*Actin was used as a control of the total parasites used in western blot. P: protein insoluble in detergent; S: detergent-soluble protein

We further investigated the role played by MCAs in the growth and replication of tachyzoites. We compared the cell division of double-knockout strains among Δku80, Δmca1 and Δmca2 using anti-r*Tg*IMC1 and anti-r*Tg*SAG1sera. The Δku80, Δmca1 and Δmca2 strains showed normal intracellular divisions with IMC1 expression and a typical rosette pattern of tachyzoites in PVs (Fig. 3a). However, Δmca1Δmca2 showed abnormal growth that was characterized by disordered tachyzoite segregation, with only 38.1% of PVs exhibiting a typical rosette organization compared to more than 90% of PVs in the other strains. IMC1 was absent in many tachyzoites of Δmca1Δmca2, which had multiple nuclei. GAP45 (gliding-associated protein 45), anchored to IMC, is responsible for motility as a component of the glideosome. IFA showed that GAP45 was also aberrantly localized in the double-knockout strain as was IMC1 (Fig. 3b). Δmca1mca2 showed many empty vacuoles in the parasites and the pellicle was composed of three membranes (a plasmalemma and two closely applied membranes that form an inner membrane complex) that were almost indistinguishable by transmission electron microscopy. In contrast, Δku80 grew normally and the three-layer membranes were clearly visible (Fig. 3c). Although the IMC1 formation of Δmca1mca2 was abnormal, it did not influence the nucleus division. The localization of SAG1 and GRA1 in Δmca1Δmca2 was the same as in the wild type (Additional file 2: Figure S2), indicating that MCA1 and MCA2 do not affect the outer membrane and PVs formation. These results suggest that *Tg*MCA1 and *Tg*MCA2 play an important role in parasite endodyogeny, especially for IMC formation.

Proteolysis of *Tg*IMC1 occurs during the maturation of *T. gondii* daughter cells, which makes the IMC1 protein detergent-resistant. We determined whether *Tg*IMC1 in Δmca1Δmca2 was detergent-soluble or resistant using 1% DOC. Western blot showed that the IMC1 in Δmca1Δmca2 was partially soluble in detergent, whereas the IMC1 in Δku80, Δmca1 and Δmca2 could not be extracted by DOC, that is, detergent-resistant (Fig. 3d). It can be concluded that loss of both MCA1 and MCA2 affects IMC1 maturation and impairs daughter cell formation.

### Tachyzoites lacking MCA1 and MCA2 were more readily induced into bradyzoites in vitro than RH and formed cysts in vivo

**Fig. 4:**
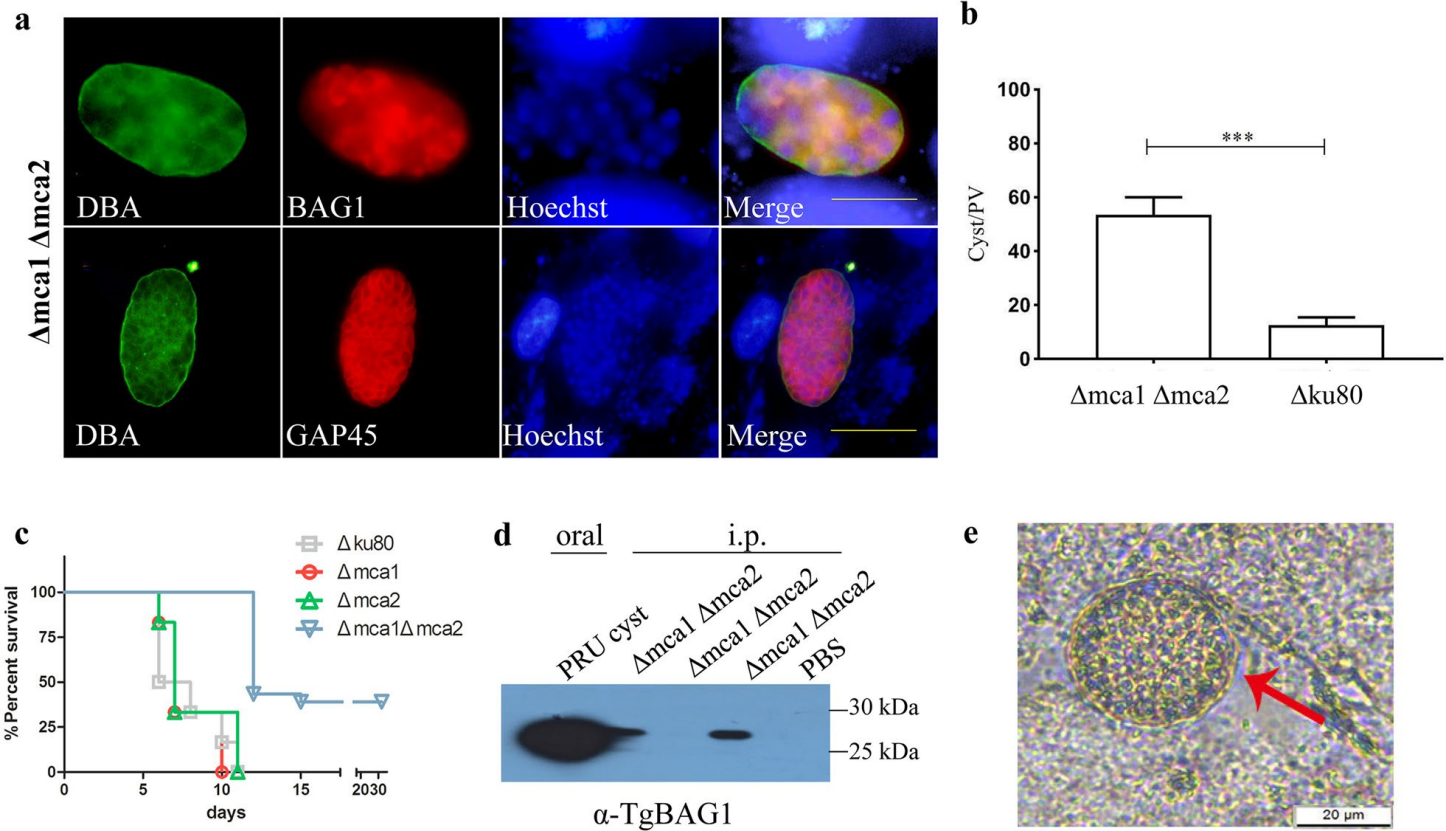
Cyst formation in vitro and virulence assay in mice. **a** Double-knockout strain and Δku80 were cultured in alkaline medium at pH 8.2 to induce cyst formation. Four days after induction, both DBA and BAG1 were detected in Δmca1mca2 by IFA (both top and bottom panels). **b** The rate of bradyzoite formation of Δmca1Δmca2 was significantly higher than that of Δku80 when cultured in alkaline medium at pH 8.2. Total 100 PVs with anti-GAP45 serum labeling parasites were counted in each strain and the number of PVs with DBA positive parasites was counted to calculate the rate of bradyzoite formation. **c** BALB/c mice were injected intraperitoneally with 100 tachyzoites of Δmca1Δmca2, Δmca1, Δmca2, or Δku80 (*n* = 5), and three independent experiments were performed with similar outcomes. Mice infected with Δmca1Δmca2 survived longer time than those infected with Δmca1, Δmca2 or Δku80, and a total of five mice of each of the three independent experiments (total 15 mice) were alive at 32 days post-infection. **d** Brain tissue of three surviving mice at 32 days post-infection was tested for the specific surface antigen protein BAG1 in bradyzoites by western blot. The brains of mice orally infected with PRU cysts were used as a positive control and mice injected intraperitoneally with PBS as a negative control. **e** The cyst was clearly observed in the brains of mice infected with tachyzoites of Δmca1Δmca2 intraperitoneally at 32 days post-infection under the microscopy. Scale bar is 20 μm

The slower the replication of *T. gondii*, the greater the chances of successful in vitro induction of tachyzoites into bradyzoites, such as in *T. gondii* type II or III strains. Since the growth of the double-knockout strain was significantly reduced, we sought to investigate the in vitro induction of tachyzoites into bradyzoites among the mutant strains. After 4 days of culturing in HFF with high pH medium, both dolichos biflorus agglutinin (DBA) and BAG1 (related bradyzoite-specific surface protein) were detected in Δmca1Δmca2 (Fig. 4a); however, most of the parasites with Δmca1, Δmca2, or Δku80 were released for 4 days of culturing in the same medium due to rapid replication. The average rate of bradyzoite formation was 53.3% for Δmca1Δmca2 and 12.3% for Δku80 based on three independent assays (Fig. 4b). We further investigated the virulence of the double-knockout strain in mice. Mice were injected intraperitoneally with 100 tachyzoites of Δmca1, Δmca2, Δmca1Δmca2 and Δku80, and we found that either Δmca1, Δmca2, or Δku80 died within 6 to 11 days. In contrast, 33% of mice (5/15) infected with the double-knockout strain survived (Fig. 4c). At 32 days post-infection, all surviving mice were sacrificed. The sera of all surviving mice were analyzed for the presence of anti-*T. gondii* antibodies and all samples were seropositive (data not shown), which indicated the success of the infection. Surprisingly, we detected *Tg*BAG1 protein in the brain tissue of part of the surviving mice (Fig. 4d). We suspect that there may be live parasites in the brain and that tachyzoites of Δmca1Δmca2 may have been transformed into bradyzoites. Mice were again infected intraperitoneally with 100 tachyzoites of Δmca1Δmca2, and brain tissue homogenate smears of surviving mice in the Δmca1Δmca2 group at 32 days post-infection were prepared, and cysts were found microscopically (Fig. 4e), although the rate of cyst formation was not high for slow-growing strains, as exemplified by type II and type III strains. In summary, parasites lacking both MCA1 and MCA2 in type I *T. gondii* RHΔku80 were defective in growth and virulence in mice and were able to convert from tachyzoites to bradyzoites, which could form cysts in the host brain.

## Discussion

Metacaspases are caspase-like proteins in non-metazoans, such as protozoa, and share common structural features of caspases, containing the His-Cys catalytic dyad domain [13]. Metacaspases can also mediate apoptosis-like cell death, as do caspases [20, 21]. *Toxoplasma gondii* undergoes apoptotic cell death in a metacaspase-dependent manner [12]. Deletion of MCA1 from *T. gondii* has no effect on the growth and virulence of the parasites. In our current study, we found that MCA2 knockout mutants also grew normally in vitro and maintained the same virulence in mice as that of RHΔku80. However, knockout of MCA1 and MCA2 severely impaired the growth and virulence of the parasites. We suspect that MCA1 and MCA2 are perhaps functionally compensatory and that once both are lost, tachyzoite growth, proliferation and virulence are seriously affected, though we previously illustrated that the genes for MCA1 and MCA2 are located on different chromosomes of the parasites, with no more than 10% sequence similarity between amino acids [12].

According to ToxoDB, the transcriptional quantities of MCAs in *T. gondii* are lower than those of most proteins, such as secreted factors or outer membrane proteins. Yeast metacaspase YCA1 in *Saccharomyces cerevisiae* has also been reported to be a protein with low expression levels [18]. During the process of MCA1 localization analysis, we found that MCA1 was distributed in the cytoplasm of *T. gondii* by IFA using an anti-*Tg*MCA1 polyclonal antibody diluted at 1:50 [12]. The serum dilution was a bit high and could cause a non-specific reaction. We constructed overexpression mutant strains to increase the amount of expression to further study the localization of MCA1 and MCA2. To avoid the nonspecific reaction of IFA, HA-tag was inserted into the coding sequences of MCA1 and MCA2, respectively. The results revealed that MCA1 and MCA2 are punctate aggregates throughout the cytoplasm of the parasites, localized in the outer membrane of the intracellular parasites. We also found that partial punctate aggregates of MCA1 and MCA2 were co-localized with IMC1, implying that MCAs may be involved in the endodyogeny of tachyzoites.

Overexpression of metacaspases in *Leishmania major* results in growth arrest due to defective cell nuclear division [19]. This phenomenon is nearly identical to that of MCA1 or MCA2 in *T. gondii*. We previously found that the proliferation of *Tg*MCA1 overexpression strains was reduced. Moreover, we found that overexpression of *Tg*MCA2 may lead to growth arrest of *T. gondii*. Knockdown of MCA2/3/5 resulted in severe growth retardation and cell cycle defects of *Trypanosoma brucei* [22]. Similarly, we found that knockout of both MCA1 and MCA2 severely impaired the proliferation and virulence of *T. gondii*. This effect could be due to IMC1 deficiency caused by the double MCA1/MCA2 knockout. First, MCA1 and MCA2 were co-localized with IMC1, implying that MCA1 may be associated with IMC1 synthesis. Second, the expression and localization of IMC1 was abnormal when both MCA1 and MCA2 were absent in intracellular tachyzoites. A large number of intracellular parasites did not exhibit rosette patterns, and IMC1 in some of the intracellular tachyzoites disappeared, with one parasite exhibiting multinucleation. *Toxoplasma gondii* replicates by a highly rigorous and unusual process, using IMC as a scaffold to assemble daughter cells within the mother cell [23]. IMC formation is dynamic, and several factors have been shown to be essential for IMC biogenesis, transportation and daughter cell maturation [24–26]. Furthermore, we confirmed that the cysteine proteases MCA1 and MCA2 are involved in IMC1 maturation. This is because immature and defective IMC1 affects the proliferation of the double-knockout mutant, but it is not clear in our assay whether the relationship between MCAs and IMC1 is direct or indirect. We also found that some extracellular Δmca1Δmca2 tachyzoites had a distorted and circular morphology, and the abnormally morphological tachyzoites exhibited weaker gliding and circling motility. However, to our surprise, the invasion and egress of the double-knockout strains were not significantly altered, which is contrary to the weak motility. Upon analysis, we found that the weak motility mainly occurred in morphologically abnormal parasites, rather than in normal parasites. When the number of each strain of infected cells was counted for invasion or egress assays, the abnormal parasites were not calculated into the total amount because we could not distinguish whether they were tachyzoites or cell debris. We speculate that the normal morphological parasites have the same ability to invade and egress as Δku80, and that the invasion and egress assays in our recent study could not respond to reality. IMC also provides the motility, invasion capacity and shape integrity of parasites [27]. GAP45, as a glideosome-associated protein, is anchored to IMC and abnormally distributed in the MCA1/MCA2 double-knockout strains, and we speculate that the reduced motility of distorted tachyzoites may be related to the defective glideosome-associated protein.

Although successful induction of type I strains of bradyzoites in vitro has been reported in a small number of studies [28–30], tachyzoites with strong virulence to mice, as exemplified by type I strains, replicate too fast and are more difficult to induce bradyzoites in vitro compared to type II and III with comparative slow replication [28], and less virulent strains are able to form cysts in the brains of mice (e.g., type II strains ME49 and PRU, or type III strains 76 K and VEG). In our study, all transgenic parasites were constructed based on RH. Although the virulence of the parental strain was strong, the virulence of Δmca1Δmca2 was significantly reduced and formed cysts not only in vitro with high pH medium but also in vivo, suggesting that the double-knockout mutant can establish chronic infection in mice.

## Conclusion

In conclusion, our findings indicate that MCAs are critical for the proliferation and virulence of *T. gondii,* which contributes to IMC1 maturation and endodyogeny. The double-knockout strain was able to develop bradyzoites in the brains of mice and establish chronic infection.

## Supplementary Information

**Additional file 1: Figure S1.** The strategy of construction of *Tg*MCA2 knockout strain. **a** Schematic illustration of the MCA2 knockout strategy and oligonucleotides used in the PCR analysis of the knockout mutant. Knockout vector (PDHFR-CD *Tg*MCA2 KO) was constructed to target complete *Tg*MCA2 gene, and genomic PCR analysis of Δmca2 confirmed the absence of MCA2 gene. P1P2 and P3P4 were used to amplify 5′ and 3′ flanking sequence of *Tg*MCA2, P5P6 was used to amplify parts of coding sequence of *Tg*MCA2. **b**, **c** and **d**, PCR and western blot were performed to confirm *Tg*MCA2 was completely knockout.

**Additional file 2: Figure S2.** The localization of surface antigen (SAG1), actin and dense granule protein (GRA1) in double knockout strain. The IFA was performed to investigate whether the outer membrane and parasitophorous vacuoles of double knockout strain had altered. **a**–**c** showed there was no significant difference between Δku80 and Δmca1Δmca2 on SAG1, actin and GRA1. Loss of MCA1 and MCA2 did not influence the parasite outer membrane and PVs. The scale bar is 5 μm.

## Data Availability

Data supporting the conclusions of this article are included within the article and its Additional files.
