# Supplementary figures and images for: Requirement of Toxoplasma gondii metacaspases for IMC1 maturation, endodyogeny and virulence in mice
Source: Parasit Vectors. 2021 Aug 12;14:400. doi: 10.1186/s13071-021-04878-0 (PMC8359067; doi:10.1186/s13071-021-04878-0)

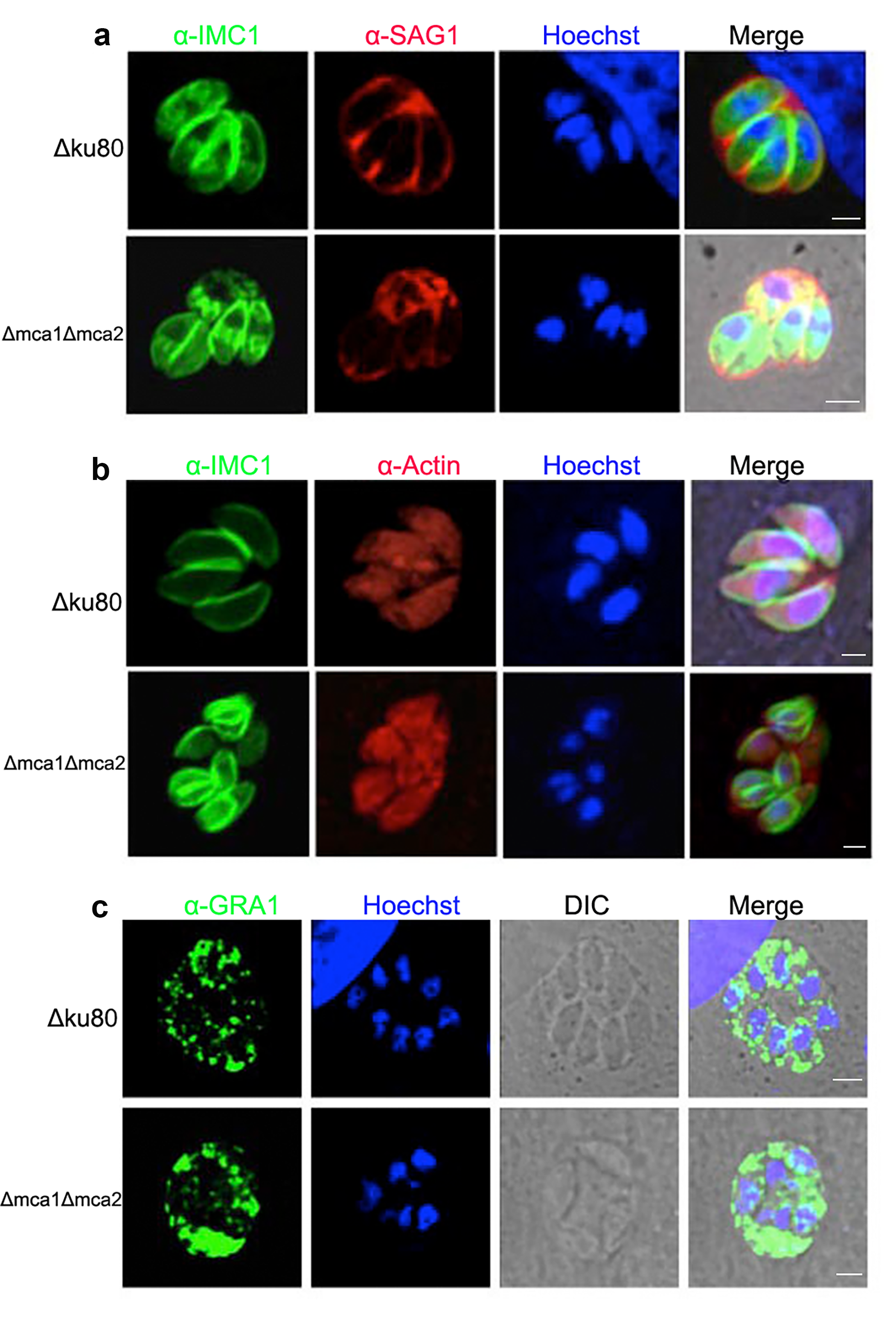

Supplement: Supplementary file 2 — Additional file 2: Figure S2. The localization of surface antigen (SAG1), actin and dense granule protein (GRA1) in double knockout strain. The IFA was performed to investigate whether the outer membrane and parasitophorous vacuoles of double knockout strain had altered. a–c showed there was no significant difference between Δku80 and Δmca1Δmca2 on SAG1, actin and GRA1. Loss of MCA1 and MCA2 did not influence the parasite outer membrane and PVs. The scale bar is 5 μm. [file 13071_2021_4878_MOESM2_ESM.tif]
